# Acute Hemichorea in an Elderly Patient With Positive Anti-centromere Antibodies and Lung Tumor

**DOI:** 10.7759/cureus.56448

**Published:** 2024-03-19

**Authors:** Koji Obara

**Affiliations:** 1 Neurology, Yasumi Hospital, Morioka, JPN

**Keywords:** tiapride, paraneoplastic neurological syndromes, chorea, autoimmune chorea, anti-centromere antibodies

## Abstract

Though rare, autoimmune paraneoplastic and non-paraneoplastic chorea can be leading causes of adult-onset acute/subacute chorea. Here, we report a case of acute-onset chorea with suspected autoimmune-mediated mechanisms in a 79-year-old female who exhibited acute-onset choreiform movements on the right side of her body. She tested positive for anti-centromere antibodies (ACAs) without displaying symptoms of scleroderma. Blood sugar levels, genetic testing for Huntington’s disease, and an antibody panel related to paraneoplastic neurological syndrome were unremarkable. Brain magnetic resonance imaging revealed no significant abnormalities. Computed tomography (CT) identified an irregularly shaped nodule in the middle lobe of the right lung. An 18F-fluoro-2-deoxy-D-glucose (FDG) positron emission tomography (PET)-CT scan showed an accumulation of radioactivity in the nodule and slight hypermetabolism in the striatum of both hemispheres. Her choreiform movements almost disappeared with a low dose of tiapride alone, without the need for anti-tumor therapy or immunotherapy. In cases of adult-onset acute/subacute chorea, investigating neoplasms and autoimmune diseases as underlying conditions is recommended. Tiapride, due to its good tolerability, is a valuable symptomatic therapy for elderly patients presenting with chorea, even in cases driven by autoimmune mechanisms.

## Introduction

As Huntington’s disease (HD) exemplifies, chorea is characterized by brief, abrupt, irregular, unpredictable, non-stereotyped movements manifesting in the face, extremities, or trunk. In cases of adult-onset acute/subacute chorea, the common causes include acute cerebrovascular diseases, such as infarction and hemorrhage, and non-ketotic hyperglycemia-induced chorea, the latter being notably prevalent among older Asian women [1]. Conversely, although rare, acute-onset chorea can also be triggered by autoimmune-mediated mechanisms, categorized into two groups in adults: paraneoplastic and non-paraneoplastic autoimmune chorea [2]. Anti-tumor therapy and immunotherapy constitute the cornerstone treatments for these types of autoimmune chorea [2-4]. Herein, we report a case of an elderly patient with acute-onset hemichorea who also tested positive for anti-centromere antibodies (ACAs) and was diagnosed with a lung tumor.

## Case presentation

A 79-year-old female visited our institution due to a one-week history of involuntary choreiform movement in her right side, including face and body weight loss of 5 kg over six months. She had no significant past medical history, including diabetes mellitus, or a family history of movement disorders or neurodegenerative diseases. Physical examination recorded a temperature of 36.6°C, a pulse of 112 beats/minute, a respiratory rate of 20 breaths/minute, and a blood pressure of 110/60 mmHg. Her skin was moist with sweat. Neurological examination demonstrated normal cognition, attention, affect, ocular movements, cranial nerves, pyramidal, sensory, and autonomic functions. She exhibited involuntary movements, such as grimacing, chewing on the right side of her face, and twisting and bending her right upper and lower limbs (Video 1).

**Video 1:** Hemichorea The patient exhibits involuntary movements, including grimacing and chewing on the right side of her face and twisting and bending her right upper and lower limbs.

These movements were irregular, worsened by mental arithmetic, and ceased during sleep. Laboratory tests were positive for antinuclear antibodies and ACAs, while other results, including blood glucose and genetic testing for HD, were unremarkable (Table 1).

**Table 1:** Laboratory testing of the patient CAG: cytosine-adenine-guanine.

Test	Results	Reference range
Fasting blood glucose (mg/dL)	99	73-109
Hemoglobin A1c (%)	4.9	4.9-6.0
Total bilirubin (mg/dL)	0.28	0.4-1.5
Aspartate aminotransferase (U/L)	25	13-30
Alanine aminotransferase (U/L)	16	7-23
Alkaline phosphatase (U/L)	108	38-113
Urea nitrogen (mg/dL)	16.5	8.0-20.0
Creatinine (mg/dL)	0.61	0.46-0.79
Creatine phosphokinase (U/L)	124	41-153
Sodium (mmol/L)	142	138-145
Potassium (mmol/L)	4.2	3.6-4.8
Chloride (mmol/L)	106	101-108
Ceruloplasmin (mg/dL)	34	21-37
C-reactive protein (mg/dL)	0.12	0-0.3
Thyroid-stimulating hormone (μIU/mL)	1.080	0.500-5.000
Free T3 (pg/mL)	2.65	2.30-4.00
Free T4 (ng/dL)	1.27	0.90-1.70
Pro-gastrin-releasing peptide (pg/mL)	80	<81
Carcinoembryonic antigen (ng/mL)	3.0	0-5
Thyroid peroxidase antibody (IU/mL)	10.4	0-15.99
Anti-glutamic acid decarboxylase antibody (pg/mL)	<5.0	0-4.9
Anti-cardiolipin IgG antibody (U/mL)	<4	0-9.9
Antinuclear antibody (titer)	1280	<40
Centromere pattern (titer)	1280	<40
Anti-centromere antibody (U/ml)	>240	<7.0
The number of CAG repeats within the HTT gene	22/19	<26

The comprehensive panel for antibodies associated with paraneoplastic neurological syndromes (BML, Inc., Saitama, Japan) was negative (Table 2).

**Table 2:** The panel of antibodies related to paraneoplastic neurological syndrome

Test	Results
AMPH	Negative
CV2 (CRMP-5)	Negative
PNMA2	Negative
Ri (ANNA-2)	Negative
Yo	Negative
Hu (ANNA-1)	Negative
Recoverin	Negative
SOX1	Negative
Titin	Negative
Zic4	Negative
GAD65	Negative
Tr (DNER)	Negative

**Figure 1:**
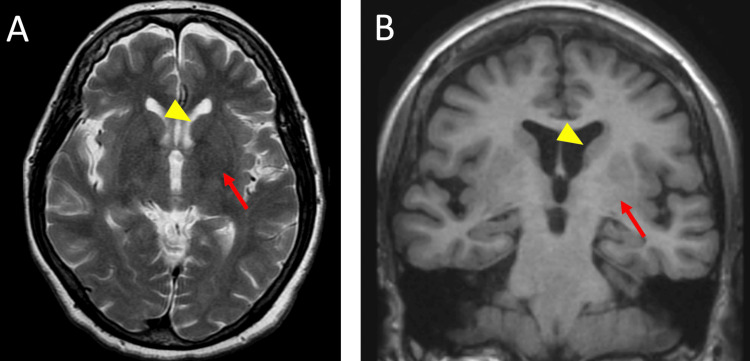
Brain magnetic resonance imaging (A) T2-weighted and (B) T1-weighted images do not show caudate nucleus atrophy or hemorrhagic and ischemic lesions in the basal ganglia. In (A) and (B), the yellow arrowhead indicates the caudate nucleus and the red arrow marks the lentiform nucleus (comprising the putamen and the globus pallidus).

Her peripheral blood smear showed no acanthocytosis. Brain MRI showed no atrophy of the caudate nucleus or lesions suggestive of vascular damage in the basal ganglia (Figure 1).

**Figure 2:**
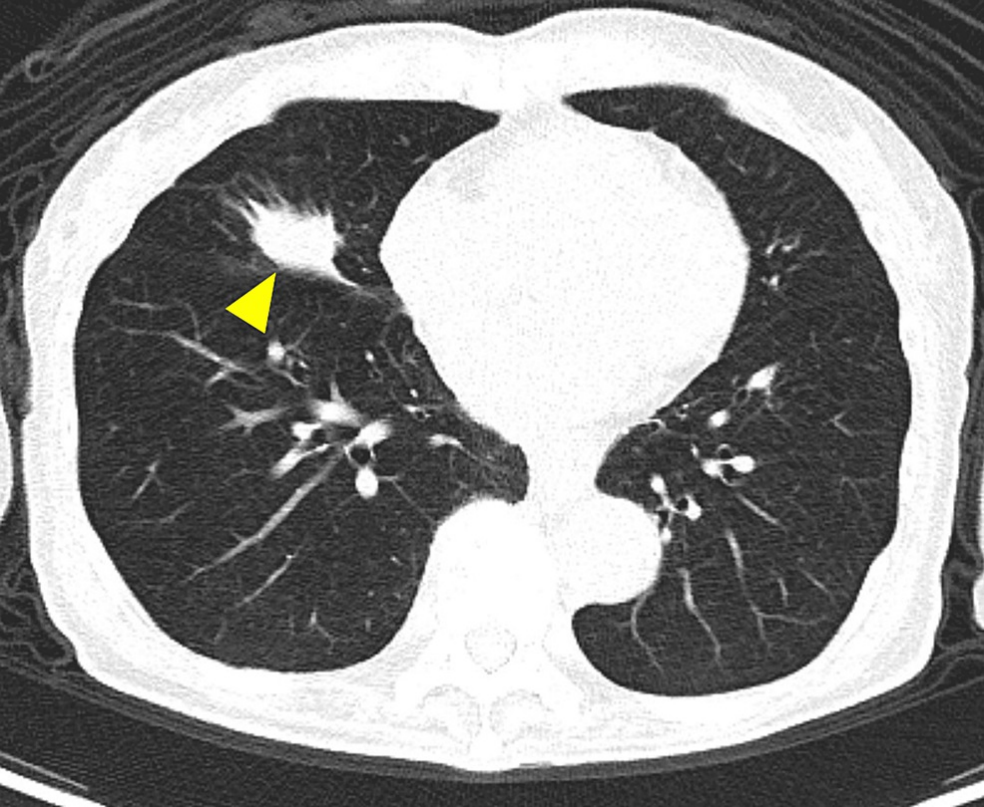
Thoracic computed tomography Thoracic computed tomography shows the irregularly shaped nodule in the middle lobe of the right lung (arrowhead).

Computed tomography identified an irregularly shaped nodule in the middle lobe of the right lung (Figure 2).

**Figure 3:**
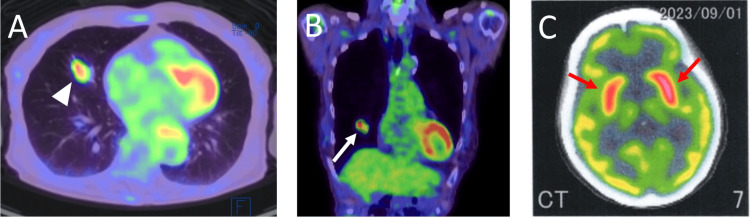
18F-fluoro-2-deoxy-D-glucose (FDG)-positron emission tomography (PET)-CT scan (A, B) Radioactivity is accumulated in the nodule in the middle lobe of the right lung (A, arrowhead; B, arrow). (C) There is slight hypermetabolism in the striatum of both hemispheres (red arrows).

An ^18^F-fluoro-2-deoxy-D-glucose (FDG) positron emission tomography (PET)-CT scan revealed increased radioactivity in the middle lobe nodule of the right lung and slight hypermetabolism in the striatum across both hemispheres (Figure 3).

Suspecting a malignant lung tumor based on CT and PET findings, she declined further diagnostic tests for her lung tumor, which remained untreated. She also did not receive immunotherapy, as, despite being ACA-positive, she showed no symptoms of scleroderma or systemic inflammation. She was treated symptomatically with tiapride 75 mg three times daily. A few days after starting tiapride, her choreiform movements significantly diminished, almost disappearing three months after beginning treatment (Video 2).

**Video 2:** Three months after taking tiapride The patient’s choreiform movements disappear.

## Discussion

We report on an elderly female with acute-onset hemichorea, who also exhibited a lung tumor suspected to be malignant and tested positive for ACAs. Her brain MRI showed no signs of cerebrovascular disease. Furthermore, her blood glucose and hemoglobin A1c levels fell within normal ranges. Therefore, we suggest that autoimmune mechanisms related to the lung tumor and ACAs could explain her choreiform movements. The literature classifies autoimmune chorea in adults into paraneoplastic and non-paraneoplastic categories [2,4]. Most often, small-cell lung carcinoma is associated with paraneoplastic chorea. Other neoplasms like non-small cell lung carcinoma, hematologic malignancies, and various adenocarcinomas are also commonly associated with it [2,4]. Finding multiple neoplasms is not rare [4]. Chorea can appear months or years before the neoplasm is diagnosed [4]. The most commonly identified antibodies in paraneoplastic chorea are CV2 (CRMP-5) and Hu (ANNA-1) IgG [2-4]. Our patient lacked anti-CV2 and Hu antibodies. However, the absence of paraneoplastic antibodies does not rule out a paraneoplastic origin, especially considering cases with antibodies beyond our panel and paraneoplastic chorea without detectable antibodies [5].

Autoimmune non-paraneoplastic chorea includes idiopathic forms and those associated with systemic autoimmune diseases [2,4]. Idiopathic forms are linked to neurological syndromes with neuronal antibodies like GAD65, CASPR2, and LGI1 [2], though chorea rarely emerges as the primary or only symptom [2]. Systemic autoimmune diseases like systemic lupus erythematosus and primary antiphospholipid syndrome often report chorea [2]. ACAs, targeting the chromosome’s centromere component in the nucleus, tested positive in our patient, a common finding in patients with limited systemic scleroderma [6]. In our search for English-language articles on PubMed, we found none describing the relationship between chorea and ACAs. However, some reports have linked cerebrovascular diseases with systemic scleroderma [7-9]. Therefore, we cannot rule out the possibility that choreiform movements may emerge due to cerebrovascular diseases in patients with systemic scleroderma. Nevertheless, our patient exhibited no symptoms of scleroderma, nor did her brain MRI show any cerebrovascular damage. The connection between her lung tumor, ACAs, and chorea remains unclear, but we recommend investigating neoplasms and autoimmune diseases as potential causes in adult-onset acute/subacute chorea.

For paraneoplastic chorea cases, targeting the underlying neoplasm with tumor resection and chemotherapy proves crucial [2-4]. On the other hand, immunotherapy, including steroids, plasma exchange, intravenous immunoglobulin, and cyclophosphamide, serves as the primary treatment for autoimmune non-paraneoplastic chorea [2,4]. Paraneoplastic chorea may also benefit from optional immunotherapy [2-4]. The effectiveness of symptomatic therapy alone, like neuroleptics and anticonvulsants, in treating autoimmune paraneoplastic and non-paraneoplastic chorea remains unproven [2-4]. However, a low dose of tiapride (75 mg/day) significantly reduced our patient’s choreiform movements without the need for anti-tumor therapy or immunotherapy. Tiapride, a preferred first-generation D2 receptor antagonist in Europe for Huntington’s disease chorea, offers a classical antipsychotic option with fewer extrapyramidal side effects, making it more suitable for elderly patients compared to haloperidol, also used for chorea [10-12]. Considering some elderly patients with autoimmune chorea may not opt for or tolerate treatment for their underlying condition, tiapride offers a well-tolerated symptomatic therapy option.

## Conclusions

Our case of an elderly female with acute-onset hemichorea, positive ACAs, and a lung tumor suspected of malignancy emphasizes the need to explore neoplasms and autoimmune diseases in adult-onset acute/subacute chorea. Tiapride presents a valuable, well-tolerated symptomatic treatment for elderly patients with chorea, even when an autoimmune mechanism is suspected.
